# A new bioinformatics tool to recover missing gene expression in single-cell RNA sequencing data

**DOI:** 10.1093/jmcb/mjaa053

**Published:** 2020-09-30

**Authors:** Jingyi Jessica Li

**Affiliations:** Department of Statistics, University of California, Los Angeles, CA 90095-1554, USA

Single-cell RNA sequencing (scRNA-seq) is a burgeoning field where experimental techniques and computational methods have been under rapid evolution in the past 6 years. These technological advances have allowed biomedical researchers to identify new cell types, delineate cell sub-populations, and infer cell differentiation trajectories in various tissue samples. Among the important features extractable from scRNA-seq data, the predominant ones are individual genes’ expression levels in single cells. Most analyses require a preprocessing step that converts a scRNA-seq dataset into a count matrix, where rows correspond to cells (or genes), columns correspond to genes (or cells), and entries are counts, i.e. a count is the number of sequenced reads or uniquely mapped identifiers (UMIs) mapped to a gene in a cell. Single-cell count matrices are highly sparse; for example, a typical matrix constructed from a droplet-based dataset may have >90% of counts as zeros.

It is well acknowledged that many zero counts are non-biological due to technological factors such as RNA degradation during library preparation, polymerase chain reaction (PCR) amplification biases for non-UMI-based technologies, and limited per-cell sequencing depths. As a result, the term ‘dropouts’ is frequently used in the single-cell field to refer to false zero counts of genes that are supposedly expressed. The prevalence of zeros hinders scRNA-seq data analyses, in particular the analyses that focus on specific genes’ expression patterns across cells. To overcome the negative effects of such data sparsity on scientific discoveries, many statistical and computational methods have been developed, falling into two types: imputation methods that aim to correct or adjust dropouts (Huang et al., 2018; Li and Li, 2018) and zero-inflated modeling methods that directly account for dropouts in specific analysis tasks (Kharchenko et al., 2014; Finak et al., 2015; Pierson and Yau, 2015; Risso et al., 2018). While the zero-inflated modeling methods provide users with one-step solutions, the imputation methods endow users with greater flexibility to design analysis pipelines. [Note that there are recent debates about whether zero-inflated modeling is needed for UMI-based data (Svensson, 2020), yet it is generally acknowledged that zero inflation exists for non-UMI-based scRNA-seq data.]

Zhang and Zhang (2021) proposes a novel imputation method PBLR for scRNA-seq data by leveraging methodological advances in low-rank matrix recovery, a prosperous topic in statistics and machine learning. Compared with existing imputation methods, PBLR is unique in its consideration of cell heterogeneity and how gene expression affects dropouts. PBLR has two key stages: cell sub-population identification and gene expression imputation. First, PBLR divides a single-cell log-transformed count matrix into submatrices, each of which corresponds to either a cell sub-population and its selected genes or all the cells and the remaining genes. Second, PBLR imputes each submatrix by solving a bounded low-rank recovery problem, where each gene has an upper bound on its imputed expression levels and the upper bound is informed by its observed expression levels (i.e. log-transformed counts).

PBLR is demonstrated to outperform six existing imputation methods on multiple synthetic and real scRNA-seq datasets. The success of PBLR is attributable to its two-stage design. Its first stage identifies cell sub-populations using an ensemble approach, and this would facilitate the next stage—imputation—for the following reason. The core of scRNA-seq imputation methods is to impute a gene’s expression level in a cell by borrowing information from similar genes’ expression levels in similar cells. However, the definition of ‘similarity’ is complicated by the existence of cell sub-populations, whose proportions and similarities differ from dataset to dataset. It has been shown that defining similar cells based on a single similarity measure or clustering algorithm may work well for one dataset but not another (Lähnemann et al., 2020). Hence, the use of an ensemble approach by PBLR, to a large extent, can lead to stable cell sub-populations supported by multiple similarity measures. As a result, the search for similar genes and cells in the imputation stage would be constrained to a cell sub-population and its selected genes, which, if accurately identified, would enhance the accuracy of imputation.

Another reason for PBLR’s success is the implementation of upper bounds in the imputation stage. It has been observed that imputed expression levels may way exceed the values we expect if imputation algorithms have no constraints on imputed values (Kannan et al., 2012). PBLR circumvents this issue by placing a reasonable upper bound learned from every gene’s observed expression levels, so that the gene’s imputed expression levels would be controlled under this upper bound as much as possible. This procedure ensures that PBLR would not output imputed expression levels that are too large to be true.

In summary, Zhang and Zhang (2021) advances scRNA-seq imputation by providing a novel computational method PBLR that addresses two major drawbacks in existing methods: (i) inaccurate identification or ignorance of cell sub-populations and (ii) possibility of outputting imputed expression levels that are unreasonably large. PBLR is an effective tool for alleviating the dropout issue in scRNA-seq data, and its methodological insights are valuable to computational researchers in the single-cell field.

*[J.J.L. is supported by grants from the National Science Foundation (DBI-1846216), National Institues of Health/NIGMS (R01GM120507), Johnson & Johnson WiSTEM2D Award, Sloan Research Fellowship, and UCLA David Geffen School of Medicine W.M. Keck Foundation Junior Faculty Award.]*
